# A Computer-Aided Diagnosis System and Thyroid Imaging Reporting and Data System for Dual Validation of Ultrasound-Guided Fine-Needle Aspiration of Indeterminate Thyroid Nodules

**DOI:** 10.3389/fonc.2021.611436

**Published:** 2021-10-07

**Authors:** Xiaowen Liang, Yingmin Huang, Yongyi Cai, Jianyi Liao, Zhiyi Chen

**Affiliations:** ^1^ Department of Ultrasound Medicine, The Third Affiliated Hospital of Guangzhou Medical University, Guangzhou, China; ^2^ Department of Ultrasound, Liwan Center Hospital of Guangzhou, Guangzhou, China; ^3^ The First Affiliated Hospital, Medical Imaging Centre, Hengyang Medical School, University of South China, Hengyang, China

**Keywords:** thyroid nodule, ultrasound, computer-aided diagnosis, TI-RADS, fine-needle aspiration

## Abstract

**Purpose:**

The fully automatic AI-Sonic computer-aided design (CAD) system was employed for the detection and diagnosis of benign and malignant thyroid nodules. The aim of this study was to investigate the efficiency of the AI-Sonic CAD system with the use of a deep learning algorithm to improve the diagnostic accuracy of ultrasound-guided fine-needle aspiration (FNA).

**Methods:**

A total of 138 thyroid nodules were collected from 124 patients and diagnosed by an expert, a novice, and the Thyroid Imaging Reporting and Data System (TI-RADS). Diagnostic efficiency and feasibility were compared among the expert, novice, and CAD system. The application of the CAD system to enhance the diagnostic efficiency of novices was assessed. Moreover, with the experience of the expert as the gold standard, the values of features detected by the CAD system were also analyzed. The efficiency of FNA was compared among the expert, novice, and CAD system to determine whether the CAD system is helpful for the management of FNA.

**Result:**

In total, 56 malignant and 82 benign thyroid nodules were collected from the 124 patients (mean age, 46.4 ± 12.1 years; range, 12–70 years). The diagnostic area under the curve of the CAD system, expert, and novice were 0.919, 0.891, and 0.877, respectively (*p* < 0.05). In regard to feature detection, there was no significant differences in the margin and composition between the benign and malignant nodules (*p* > 0.05), while echogenicity and the existence of echogenic foci were of great significance (*p* < 0.05). For the recommendation of FNA, the results showed that the CAD system had better performance than the expert and novice (*p* < 0.05).

**Conclusions:**

Precise diagnosis and recommendation of FNA are continuing hot topics for thyroid nodules. The CAD system based on deep learning had better accuracy and feasibility for the diagnosis of thyroid nodules, and was useful to avoid unnecessary FNA. The CAD system is potentially an effective auxiliary approach for diagnosis and asymptomatic screening, especially in developing areas.

## Introduction

Approximately 95% of endocrine cancers involve the thyroid, which contributes to the continually increasing incidence of thyroid cancer (1, 2). Ultrasound (US) is widely used as a non-invasive and effective screening modality for the detection of thyroid nodules. However, although various diagnostic standards and guidelines are available, the diagnostic specificity remains unsatisfactory (3). Since first proposed in 2009, the Thyroid Imaging Reporting and Data System (TI-RADS) has become among the most widely applied approaches for US-based diagnosis of thyroid diseases, as this system allows the physician to analyze the composition, echogenicity, shape, orientation, margin, calcification, presence of a halo, and type of vascularization for more accurate diagnosis and treatment (4). In 2017, the American College of Radiology launched the final version of the TI-RADS, which uses a scoring method to optimize and standardize US-guided fine-needle aspiration (FNA) (5). However, this modality is still limited by subjectivity and inconsistencies when applied clinically. The specificity of FNA for the detection of thyroid nodules is reportedly only 60–70%, suggesting a high occurrence of non-diagnostic results (6, 7). Moreover, since there is currently no consensus on the standardization of US-guided FNA (8), it is difficult to unify all physicians to follow the same diagnostic criteria, even within the same department or hospital. The use of TI-RADS has changed the focus of diagnostic subjectivity to the detection of features, which can decrease but not completely avoid intra-observer variability (9). Therefore, a consistently effective and accurate method for the diagnosis of thyroid nodules with good repeatability is urgently needed.

Over the past decade, artificial intelligence (AI)-aided US techniques, which integrate US and computer science, have become increasingly employed for the detection and diagnosis of thyroid diseases (10, 11). From traditional machine learning to deep learning methods, many algorithms, such as the Support Vector Machine (12), GoogleNet (13), and a convolutional neural network (14), have been shown to be effective for US-based diagnosis of thyroid nodules. These advanced methods have become increasingly used in recent years due to advances in commercial software applications, such as AmCad-UT (AmCad BioMed Corporation, Taipei City, Taiwan) (15), S-Detect (Samsung Medison Co., Ltd., Seoul, Korea) (3), and AI-SONIC (Demetics Medical Technology, Zhejiang, China). AI-SONIC is a fully automatic diagnosis system based on deep learning, which includes a training set consisting of more than 60,000 US images of the thyroid. A cascade convolutional neural network (CNN) is a type of hybrid deep learning model based on a special splitting method and two different CNN architectures (one with 15 convolutional layers and two pooling layers for segmentation, and another with four convolutional layers and four pooling layers for detection). In this study, 10-fold cross-validation was performed with a training set, validation set, and testing set ratio of 8:1:1. The detection and diagnostic efficiency of AI-SONIC are reportedly very good, as evidenced by a Dice score of 0.9224 with a diagnostic area under the curve (AUC) of 0.98 (16, 17). Besides the excellent diagnostic accuracy, other advantages of this software include an automatic recommendation of a follow-up plan, including FNA, and the provision of detailed information about the features of the thyroid detected by the software itself to render the recommendations more understandable and acceptable. However, as far as we know, there is no report on the interpretability of CAD in thyroid ultrasound diagnosis, and the ability of CAD in improving novices’ diagnostic efficiency based on AI-SONIC needs to be proved.

In our center, a dual-verification process is used to improve the reliability of the results. In order to validate the use of this approach in clinical practice in areas lacking medical resources, the diagnostic efficiency was compared among an expert, a novice, and a computer-aided diagnosis (CAD) system. Furthermore, the relationship between the pathology results and US features detected by the CAD system were analyzed to determine whether the CAD system can improve the efficiency of US-guided FNA.

## Materials and Methods

### Patients

A total of 138 thyroid nodules were collected from 124 patients who received treatment at the Third Affiliated Hospital of Guangzhou Medical University and Liwan Center Hospital of Guangzhou from January 2016 to May 2019. The final diagnosis of the thyroid nodules was confirmed with the use of specimens collected during surgery or FNA. Detailed patient data was retrieved from the Hospital Information Manage System. The inclusion criteria were (1) complete and clear US images before surgery or FNA and (2) the inclusion of a single nodule in a two-dimensional US image, while the exclusion criteria were (1) a lack of pathology results, (2) a history of partial thyroidectomy, and (3) any complication of a diffuse thyroid disease. The study protocol was approved by the local ethics committee, and written informed consent was obtained from all patients prior to study inclusion.

### Equipment and Data Analysis

The US examinations were performed with the use of different brands of equipment, which included the Phillips IU22/IE33/CX50 systems (Philips Healthcare, Eindhoven, Netherlands), Hitachi Hi Vision Preirus/Ascendus systems (Hitachi Ltd., Tokyo, Japan), GE Logiq E9/S6/S8/E6/E8 systems (GE Healthcare, Milwaukee, WI, USA), Siemens S1000/S2000 systems (Siemens Healthineers, Munich, German), Resona 7 (Mindary, Shenzhen, China), and Aplio 300/500 systems (Toshiba Corporation, Tokyo, Japan). An expert with 8 years of experience and a novice with 1 year of experience in US examinations of the thyroid reviewed the nodules and diagnosis recommended by the TI-RADS, respectively, and recorded the final score of the nodules. To ensure the validity of the assessments, the score of the TI-RADS as judged by the expert was confirmed by another expert with 15 years of experience.

### Detection and Diagnosis With the CAD System

**Figure 1:**
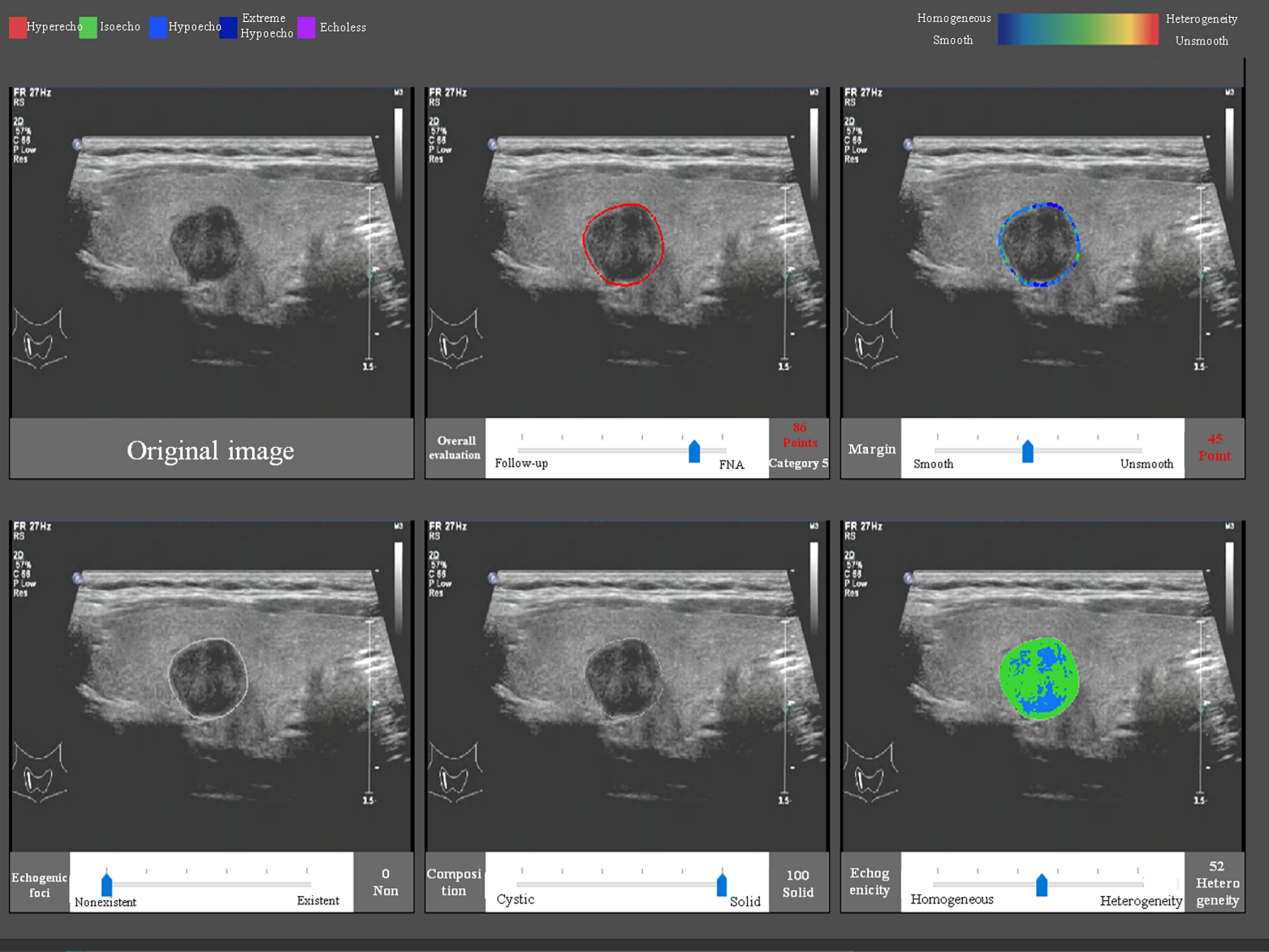
The CAD interface for detection and diagnosis of thyroid nodules. Upper row (from left to right): original image, overall evaluation (score and category), and margin (score and category). Bottom row (from left to right): echogenic foci (percentage and classification), composition (percentage and classification), and echogenicity (percentage and classification).

The CAD software was loaded into a computer, and the US images were uploaded into the software. After confirmation that there was only one nodule on the center of the screen, the software could detect the nodule automatically and quantitatively analyzed the margin, composition, echogenicity, and the existence of echogenic foci (**Figure 1**). This process only took 1–2 s. If the CAD system failed to identify a nodule, the physician defined the region of interest manually and the analysis was repeated. The probability of a malignant *versus* benign nodule and a recommendation of a follow-up were provided. Furthermore, the software would output a six-in-one interface, which included information of the shape, margin, composition, echogenicity, and the existence and degree of echogenic foci, which were used to apply the results systematically.

### Recommendation of FNA

**Figure 2:**
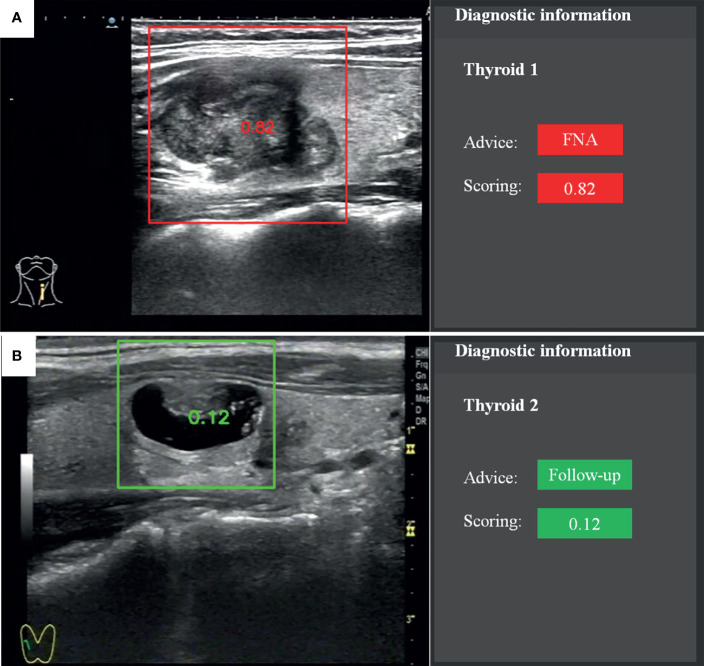
Recommendation and scoring provided by the fully automatic CAD system. **(A)** Papillary thyroid carcinoma. **(B)** Nodular goiter.

The recommendation of FNA by the TI-RADS was based on the following classification criteria of thyroid nodules: 0 points, TR1; 2 points, TR2; 3 points, TR3; 4–6 points, TR4; and ≥7 points, TR5. A score of >4 points indicated a 5–20% chance of malignancy. TR1–2 indicated no need for FNA. If the largest diameter of a TR3 nodule was ≥1.5 cm, TR4 nodule ≥1.0 cm, or TR5 nodule ≥0.5 cm, the patient should be followed up. If the largest diameter of a TR3 nodule was ≥2.5 cm, TR4 nodule ≥1.5 cm, or TR5 nodule ≥1.0 cm, FNA was needed. The CAD system provided advice and scoring according to the probability value (**Figure 2**). The cytological results of FNA and histological results of surgery were considered as reference standards.

### Statistical Analysis

All statistical analyses were performed using IBM SPSS Statistics for Windows, version 25.0. (IBM Corporation, Armonk, NY, USA). Quantitative data are expressed as the mean ± standard deviation. The pathology results were regarded as the gold standard. Receiver operator characteristic curve analysis was performed to evaluate the diagnostic performance and compared in terms of sensitivity, specificity, positive predictive value (PPV), negative predictive value (NPV), and accuracy. The AUCs were computed to assess the diagnostic performances of the CAD system, while performance of the TI-RADS was assessed by the expert and novice. Paired comparisons of sensitivity and specificity were evaluated using the chi-squared test and the McNemar test. A two-sided probability (*p*) value of <0.05 was considered statistically significant.

## Results

### Population Characteristics

Of the 138 thyroid nodules collected from 126 patients (including 27 males and 99 females), 56 were malignant (54 papillary thyroid carcinomas, one follicular thyroid carcinoma, and one indeterminate malignant tumor) and 82 were benign (79 nodular goiters and three adenomas). The mean age of the patients was 46.4 ± 12.1 (range, 12–70) years.

### Analysis of Diagnostic Accuracy

**Figure 3:**
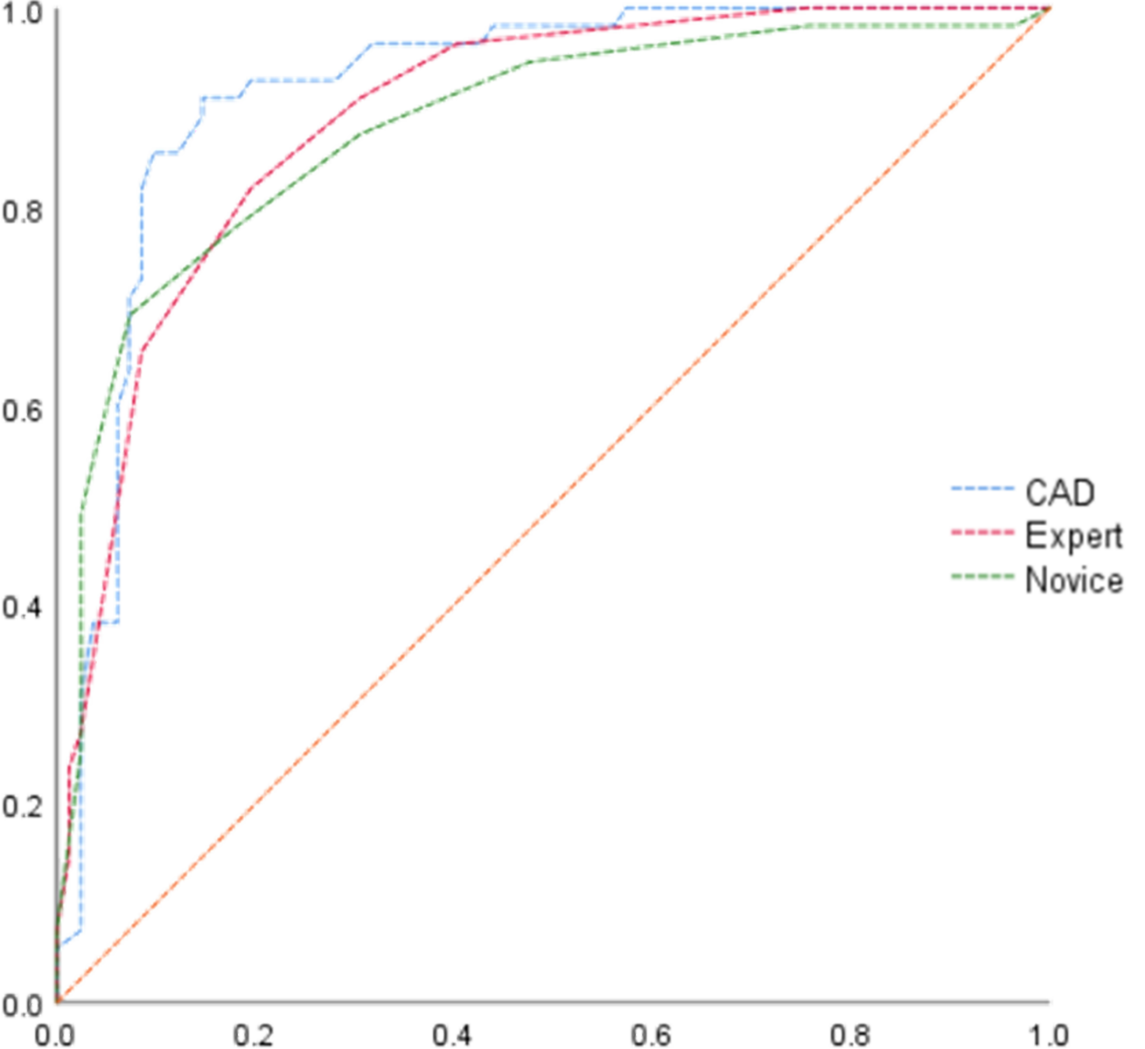
ROC curves of the CAD system, expert, and novice for US diagnosis of thyroid nodules.

The diagnostic accuracy of the CAD system, expert, and novice is shown in **Table 1**. The results show that the CAD system had better diagnostic sensitivity, specificity, PPV, NPV, and accuracy than the expert and novice (*p* < 0.05). The AUCs of the CAD system, expert, and novice were 0.919, 0.891, and 0.877, respectively (**Figure 3**).

**Table 1:** Diagnosis accuracy of CAD system, expert and novice in the study.

	Sensitivity	Specificity	PPV	NPV	Accuracy
CAD system	0.642	0.930	0.929	0.676	0.761
Expert	0.605	0.923	0.929	0.585	0.725
Novice	0.565	0.913	0.929	0.512	0.681

PPV, Positive Predictive Value; NPV, Negative Predictive Value.

### Correlations Among Features Detected by the CAD System and the Pathology Results

To validate the diagnostic efficiency of features detected by the CAD system, differences in the features of echogenic foci, margin, composition, and echogenicity between benign and malignant nodules were analyzed (**Table 2**). The results revealed significant differences in the existence of echogenic foci and the echogenicity of the nodules between groups (*p* < 0.05).

**Table 2:** Correlation among diagnostic features detected by CAD and pathology result.

Diagnostic features		Benign	Malignant	*P* value
Echogenic foci	Existent	40	17	0.031^*^
	Nonexistent	42	39	
Margin	Smooth	26	18	0.957
	Unsmooth	56	38	
Composition	Cystic (or almost)	0	3	0.065
	Solid (or almost)	82	53	
Echogenicity	Homogeneous	10	17	0.008^*^
	Heterogeneity	72	39	

^*^P < 0.05.

### Efficiency of FNA Recommendation

The accuracy of a recommendation of FNA was compared between the expert (using TI-RADS) and the CAD system (**Table 3**). According to the diagnoses of the expert, there were five TR1-2 nodules, which did not require follow-up or FNA. Corresponding to the recommendations of the TI-RADS (i.e., TR3 nodule ≥2.5 cm, TR4 nodule ≥1.5 cm, and TR5 nodule ≥1.0 cm), of a total of 60 thyroid nodules, 31 were benign and 29 were malignant. According to the results obtained with the CAD system, FNA was recommended for a total of 58 thyroid nodules (10 benign and 48 malignant). Hence, there were significant differences between the expert and CAD system (*p* < 0.001).

**Table 3:** FNA recommendation by expert (using TI-RADS) and CAD system.

Recommendation	Groups	Total	Benign	Malignant
FNA	ACR TI-RADS	60	31	29
	CAD system	58	10	48
Follow-up	ACR TI-RADS	78	51	27
	CAD system	80	72	8

FNA, Final-needle aspiration.

## Discussion

Since most thyroid nodules have indolent behavior with positive prognoses, patients are potentially receiving excessive care. The key point to an accurate diagnosis is to improve efficiency and avoid unnecessary FNA as much as possible (18, 19). Although AI techniques continue to rapidly develop, further investigations of efficiency and interpretability are needed before applications for the diagnosis of thyroid nodules. In this study, the efficiency of a CAD system (i.e., AI-SONIC) was validated for the diagnosis of thyroid nodules by comparisons of an expert and novice using the TI-RADS. Also, the features detected by the CAD system, which can lead to explicable results, were analyzed. Furthermore, the efficiency of the recommendation of FNA by the CAD system was investigated. It seems that the CAD system, based on deep learning, had better performance and thus is suitable as an aid for the diagnosis and screening asymptomatic thyroid nodules, especially in areas lacking medical resources.

### Performance of the CAD System for US Diagnosis of Thyroid Nodules

Regardless of the high incidence of thyroid nodules, there is a relatively low risk of malignancy. Accurate diagnosis is not only helpful for a diagnosis, but a proper recommendation of FNA can also be obtained. The first time AI was used for US-based diagnosis of thyroid nodules dates back to 1988 (20). AI techniques for US-based diagnosis of thyroid nodules can be divided into two parts: recognition (or segmentation) and classification. In recent years, various advanced algorithms have been proposed to improve the diagnostic accuracy, including support vector machines (21), random forest learning algorithm (22), and artificial neural network algorithm (23), among others. The development of deep learning is another great advancement, which has led to faster, more accurate, and fully automatic diagnoses. However, clinical application is far different from laboratory investigations, thus an objective assessment is necessary. Therefore, since there are differences in data, diagnostic criteria, and equipment, it is impractical to directly compare the diagnostic efficiency of a particular system among different studies. For example, Szczepanek-Parulska et al. compared the performance of the S-Detect System and the European Thyroid Imaging Reporting and Data System for the diagnosis of 133 cases, and found that the CAD system had better performance (3). However, in another study using the same commercial software, the results showed that the CAD system had lower specificity than an experienced radiologist (41.2 *vs* 83.5%, respectively) (24). In the present study, the sensitivity, specificity, PPV, NPV, and accuracy of the CAD system (AUC = 0.919) were higher than the expert (AUC = 0.891) and novice (AUC = 0.877), indicating that the CAD system based on deep learning could improve the diagnostic efficiency of a novice, similar to the findings of a previous study (25). However, the CAD system is more likely to be an auxiliary approach for diagnosis and asymptomatic screening, rather than a replacement of experienced experts. On the other hand, low specificity remains problematic for US-based diagnoses with a CAD system (26). Notably, in the present study, the highest specificity was only 93.0%. There are several possible explanations for this finding. First, the classification of thyroid nodules with the TI-RADS by the expert and novice was based on scoring criteria, while a diagnosis with the CAD system was closely related to the quality of the image data. Second, since this was a retrospective study, it was inevitable to avoid selecting more positive cases with high-quality images. Third, all patients in this study had undergone surgery or FNA, which may have led to selection bias. The required time for diagnosis among the groups was not analyzed, since it was obvious that the CAD system can output the results and conclusions much faster.

### Interpretability of the CAD System

With the continued applications in the medical field, the interpretability of AI is gradually playing an important role in the acceptance by clinicians (27), especially for software based on deep learning. AI-SONIC software provides detailed feature information detected automatically about the existence of echogenic foci, margin, composition, and echogenicity of the thyroid nodules. Here, analyses of the differences in features and pathology revealed that only the existence of echogenic foci and echogenicity of the thyroid nodules were relevant. Theoretically speaking, the existence of echogenic, especially punctate, foci was highly related to papillary thyroid carcinoma (28). However, the results showed that echogenic foci detected by the CAD system were more common with benign, rather than malignant, nodules, likely because spongiform-type nodules associated with goiter were not excluded. In addition, the CAD system was unable to accurately differentiate macrocalcifications from microcalcifications. Besides the failure to exclude lesions associated with nodular goiter, the insignificance of composition features might also relate to liquefactive necrosis in rapidly growing malignant tumors. Furthermore, to better validate the robustness of the CAD system, the validation set had not unified the standard plane of thyroid nodules; thus, some of the obtained images were cross-sections, while others were longitudinal sections, which could have led to uncertainties of boundaries of benign nodules. This was potentially one of the reasons why there were no significant differences in margin features between groups.

### Application of CAD in FNA Recommendation

Optimally, the diagnosis and treatment of thyroid nodules should avoid unnecessary FNA (29). Since a CAD system can reduce intra- and inter-observer variability, some studies have validated the value of a CAD system to improve the efficiency of a recommendation of FNA (30–32). In the present study, the performance of the AI-SONIC based on deep learning was much better for a recommendation of FNA than the expert using the TI-RADS (*p* < 0.001). As compared with the TI-RADS evaluated by expert, the CAD system recommended FNA for more malignant thyroid nodules and follow-up for more benign nodules. Because most of the decisions for FNA are made by experts, the efficiency of a recommendation of FNA between the CAD system and novice was not compared.

### Limitations

There were several limitations to this study that should be addressed. First, this was a retrospective study; thus, there was selection bias and a lack of standardization. Although this study was conducted in two centers, the sample size was relatively small. In addition, the features automatically detected by the software were not completely identical to those of the TI-RADS, especially the shapes of the nodules. More specific situations should be considered to further improve the CAD system, especially accurate identification of echogenic foci of malignant nodules and spongiform-type nodules associated with goiter. Lastly, intra-observer variability could not be ruled out. Hence, future studies to address these issues are warranted.

## Conclusions

The application of a CAD system is now changing the approaches for the diagnosis and treatment of thyroid nodules. In the present study, the diagnosis on thyroid nodules was improved with the CAD system, although many problems remain that must be addressed. We believe that with the continued development of technology and medical science, the CAD system based on deep leaning and larger datasets will become a suitable, rapid, and high-quality approach for US-based diagnosis and screening of thyroid nodules, especially in areas lacking medical resources.

## Data Availability Statement

The raw data supporting the conclusions of this article will be made available by the authors, without undue reservation.

## Ethics Statement

Written informed consent was obtained from the individual(s) for the publication of any potentially identifiable images or data included in this article.

## Author Contributions

XL and YH are responsible for the substantial contributions to conception and design, as well as drafting the article. XL, YC, and JL are responsible for the acquisition of data and the analysis of data. ZC is responsible for the final approval of the version to be published. All authors contributed to the article and approved the submitted version.

## Funding

This work is supported by the Research Projects of the National Natural Science Foundation of China (No. 82102054), Major Research Projects of Universities in Guangdong Province (No. 2019KZDZX1032), Youth Foundation of Scientific Research of Third Affiliated Hospital of Guangzhou Medical University (No. 2018Q18).

## Conflict of Interest

The authors declare that the research was conducted in the absence of any commercial or financial relationships that could be construed as a potential conflict of interest.

## Publisher’s Note

All claims expressed in this article are solely those of the authors and do not necessarily represent those of their affiliated organizations, or those of the publisher, the editors and the reviewers. Any product that may be evaluated in this article, or claim that may be made by its manufacturer, is not guaranteed or endorsed by the publisher.
